# Suggested Sustainable Medical and Environmental Uses of Melanin Pigment From Halotolerant Black Yeast *Hortaea werneckii* AS1

**DOI:** 10.3389/fmicb.2022.871394

**Published:** 2022-04-13

**Authors:** Asmaa Elsayis, Sahar W. M. Hassan, Khaled M. Ghanem, Heba Khairy

**Affiliations:** ^1^National Institute of Oceanography and Fisheries (NIOF), Cairo, Egypt; ^2^Department of Botany and Microbiology, Faculty of Science, Alexandria University, Alexandria, Egypt

**Keywords:** melanin, marine black yeast, natural antioxidant, antimicrobial, heavy metal removal

## Abstract

The marine ecosystem is a complex niche with unique environmental circumstances. Microbial communities from the sea are one of the main origins of compounds with tremendous capabilities. Marine yeasts have the ability to produce secondary metabolites that are architecturally distinct from those found in terrestrial species. Melanin pigment synthesized by marine halotolerant black yeast *Hortaea werneckii* AS1 isolated from Mediterranean salt lakes in Alexandria, Egypt was found to exert a radical scavenging effect on 2,2-diphenyl-1-picrylhydrazyl (DPPH) with an IC_50_ of 61.38 μg/ml. Furthermore, it showed no cytotoxicity toward human skin fibroblast cell line (HSF) with an IC_50_ value above 0.1 mg/ml. The antimicrobial capability of the pigment was revealed against the tested number of bacterial and fungal strains with the highest inhibition zone of 25 mm against *Aeromonas* sp. and a growth inhibition percentage up to 63.6% against *Aspergillus niger*. From an environmental impact point of view, the pigment disclosed a heavy metal removal efficiency of 85.7, 84.8, and 81.5% for Pb^2+^, Cd^2+^, and Ni^2+^, respectively, at 100 mg/L metal concentration. The previously mentioned results suggested melanin from *H. werneckii* AS1 as a promising biocompatible candidate in various medical, cosmetics, pharmaceutical, and environmental applications.

## Introduction

Pigments can be defined as vivid chemical compounds capable of absorbing light in the visible spectrum zone (Sajid and Akbar, 2018; Thaira et al., 2018). Pigments are categorized under two general classes: synthetic and natural. Nowadays, natural pigments are gaining popularity over synthetic ones, as participants in various applications of food, cosmetic, and pharmaceutical industries. This interest could be due to the undesirable and potentially harmful effects of synthetic dyes, since some of them were believed to be carcinogenic and causative agents of allergic responses (Kumar et al., 2011). As a result, obtaining pigments of natural origin is considered a magnificent track for research and improvement due to their excellent properties: safe, easily degradable, and environmentally friendly (Pombeiro-Sponchiado et al., 2017).

Melanins are one of the exceptional natural pigments that are harmonized by all biological kingdom’s members and have contributed to a variety of functions (Surwase et al., 2013; Kurian and Bhat, 2018; Ghadge et al., 2020). The majority of melanin’s functions are attributed to providing protection from severe environmental stimuli such as ionizing radiation, extreme temperatures, desiccation, hypersalinity, heavy metals, and radionuclides (Gessler et al., 2014; Wilson, 2016).

Diverse classes of melanins can be synthesized by different fungal species (Mattoon et al., 2021). The black yeast *Hortaea werneckii* is the ultimate acknowledged halotolerant strain in fungi, capable of growing across a wide range of NaCl concentrations from 0 to saturation at 30% NaCl, and is well-known for its capacity to naturally synthesize melanin pigment (Chung et al., 2019; Zalar et al., 2019). In the face of exceptional saline surroundings, *H. werneckii* has developed a captivating survival mechanism that includes increasing melanization by forming a stable layer of granular melanin in the cell wall’s outlying section (salt-based distributed); as a result, the size of the pores in the cell wall decreased, diminishing the leakage of its principal compatible solute, glycerol, from within the cells (Kogej et al., 2007; Zalar et al., 2019).

Taking into account the unique physical and chemical properties of melanins in addition to their important biological functions, melanins can be utilized for various medical, environmental, and biotechnological applications (Mattoon et al., 2021). Many diseases such as cancer, autoimmune diseases, and Alzheimer’s disease are caused by the destructive action of reactive oxygen species (ROS) to the structure and function of sensitive intracellular molecules (Kurian et al., 2015; Cordero et al., 2017). Unpaired electrons documented in the conserved structure of melanin pigments allow them to interact rapidly with free radicals and limit the synthesis of reactive oxygen species (Gessler et al., 2014). Also, melanin’s oxidizing and reducing moieties (o-quinone and o-hydroquinone, respectively), detected among various melanin classes, make melanins an attractive antioxidant candidate (Kim et al., 2014; Roy and Rhim, 2021). Due to the side effects of some synthetic antioxidants, melanin can be employed as efficient, ecofriendly natural antioxidant molecules (Wilson, 2016).

In addition, melanins have been shown to suppress the growth of a variety of microorganisms, including *Escherichia coli*, *Candida albicans, Streptococcus pyogenes*, methicillin-resistant *Staphylococcus aureus* (MRSA), *Bacillus subtilis*, *Erwinia carotovora*, and *Pseudomonas aeruginosa* (Zerrad et al., 2014; Laxmi et al., 2016; Saleh et al., 2018). Melanin resistance to pathogenic microbes can be traced back to the fact that it is produced as part of the immune response against microbial attack among fungi, plants, as well as invertebrates (Nosanchuk et al., 2015; Roy and Rhim, 2021).

Taking into consideration the various functional groups in melanin, such as =O, –OH, –NH, and –COOH, that have a considerable affection for metal ions, melanin can be an ideal alternative for bioremediation of toxic heavy metals polluting the environment through distinctive means, including industrial, agricultural, and waste dumping practices (Sajjan et al., 2013; Thaira et al., 2018; Manirethan, 2019). Melanin’s negative charge and large surface area facilitate the formation of ionic complexes with various metal ions (Manirethan et al., 2018). Furthermore, melanin’s acid resistance, insolubility in water as well as most organic solvents, sustainability, and biocompatibility make it an appealing substrate for evolving an eco-friendly approach for detoxifying and withdrawing metals in a variety of applications, particularly at its low concentrations (Eisenman and Casadevall, 2012; Thaira et al., 2018; Tran-Ly et al., 2020).

Multiple useful applications relying on melanin, as well as the current market’s higher cost of melanin, necessitate a thorough search for alternative sources and effective extraction techniques that ensure a relatively higher output of melanin (Ghadge et al., 2020). Microorganisms as melanin producers are seen as a cost-effective as well as environmentally friendly alternative to synthetic melanins (Ben Tahar et al., 2019; Martiínez et al., 2019).

In a previous work, melanin pigment was produced by a locally isolated promising marine halotolerant *H. werneckii* AS1 strain; the extracted pigment was characterized and the culture conditions were optimized to obtain the highest yield of melanin pigment (Elsayis et al., 2022). The current study attempts to examine the various valuable medical and environmental utilizations of this beneficial pigment.

## Materials and Methods

### Strain Isolation and Growth Conditions

*Hortaea werneckii* AS1 was isolated from sediment samples obtained from solar saltern in Egypt, exactly from Alexandria international coastal road “31.068709, 29.774321” in December 2019 (Elsayis et al., 2022). The isolate was deposited in Assiut University Mycological Center with the number (AUMC 14501). Melanin production was performed in modified Vogel liquid medium prepared with seawater (El-Batal and Al Tamie, 2016). The culture was incubated for 10 days at 30°C with an agitation speed of 180 rpm.

### Melanin Extraction and Identification

Melanin pigment extraction and purification process was carried out following the Gadd (1982) protocol with some modification. After 10 days of incubation, the culture was centrifuged at 10,000 rpm for approximately 10 min, the supernatant was discarded, and pellets were rinsed with distilled water and then collected for the extraction step. Melanin was recovered from yeast biomass by autoclaving it with 1N NaOH. In order to obtain the supernatant containing the desired melanin pigment, centrifugation of the treated biomass for about 10 min at 8,000 rpm was performed. After that, concentrated HCl was added to acidify the supernatant to pH 2 followed by a centrifugation step for 10 min at 10,000 rpm, which resulted in precipitating the melanin pigment. The extracted melanin was then dried in a dehumidified environment and characterized using ultraviolet-visible (UV-Vis) spectrophotometry, Fourier transform-infrared spectroscopy (FTIR), elemental analysis, and scanning electron microscopy (SEM) (Elsayis et al., 2022).

### Biological Impact

#### Antioxidant Activity of the Purified Melanin

The 2,2-diphenyl-1-picrylhydrazyl (DPPH) radical scavenging assay has been frequently applied to precisely measure the antioxidant activity of the compounds (Fogarasi et al., 2015). In order to identify a range within which the inhibitory concentration 50 (IC_50_ represents the effective melanin concentration required to achieve 50% inhibition) lies, solutions of the melanin pigment were prepared in dimethyl sulfoxide (DMSO) with concentrations of 100 and 1,000 μg/ml. DPPH free radical assay was performed according to Boly et al. (2016) method. In a 96-well microplate (*n* = 6), 100 μl of freshly prepared DPPH reagent (0.1% in methanol) was mixed with 100 μl of melanin concentrations (100 and 1,000 μg/ml) and then the reaction was incubated at room temperature for 30 min in dark. The resulting reduction in DPPH color intensity was measured at 540 nm using a microplate reader, the FLUOStar Omega, at the end of incubation time. As a standard, Trolox solutions ranging from 5 to 50 μM were used. The percentage of DPPH radical scavenging activity was estimated according to the following equation:


DPPHscavengingactivity(%)=[(Ac-As)/Ac]×100

Where *Ac* is the absorbance of control and *As* is the absorbance of sample. Data were analyzed using *Microsoft Excel* and the IC_50_ value was calculated using *GraphPad Prism 5* (Chen et al., 2013).

#### Assessment of the Purified Melanin Cytotoxicity and *in vitro* Anticancer

Cell viability was assessed by Sulforhodamine B (SRB) assay (Skehan et al., 1990). Human Skin Fibroblast cell line (HSF), Hepatocellular carcinoma cell line (HepG2), and Breast Adenocarcinoma cell line (MCF-7) were obtained from Nawah Scientific Inc. (Mokattam, Cairo, Egypt). Cells were maintained in Dulbecco’s Modified Eagle medium (DMEM) supplemented with 100 mg/ml of streptomycin, 100 units/ml of penicillin, and 10% of heat-inactivated fetal bovine serum in a humidified, 5% (v/v) CO_2_ atmosphere at 37°C. Aliquots of 100 μl of cell suspension (5 × 10^3^ cells) were inoculated in 96-well tissue culture plate and incubated in complete media for 24 h. Cells were treated with another aliquot of 100 μl media containing melanin at various concentrations (0.01, 0.1, 1, 10, and 100 μg/ml). After 72 h of exposure, cells were fixed by replacing media with 150 μl of 10% trichloroacetic acid (TCA) and incubated at 4°C for 1 h. The TCA solution was removed, and the cells were washed five times with distilled water. Aliquots of 70 μl of SRB solution (0.4% w/v) were added and incubated in a dark place at room temperature for 10 min. Plates were washed 3 times with 1% acetic acid and allowed to air-dry overnight. Then, 150 μl of TRIS (10 mM) was added to dissolve protein-bound SRB stain; the absorbance was measured at 540 nm using a BMG LABTECH-FLUOStar Omega microplate reader (Ortenberg, Germany) (Allam et al., 2018). Cell viability was quantified according to the following formula:


Viability(%)=(Absorbanceoftestsample/Absorbanceofcontrol)×100.

#### *In vitro* Wound Healing Assay

The wound healing assay was performed on the Human Skin Fibroblast cell line (HSF), obtained from Nawah Scientific Inc. (Mokattam, Cairo, Egypt). Cells were plated at a density of 3 × 10^5^/well onto a coated six-well plate for scratch wound assay and cultured overnight in 5% FBS-DMEM at 37°C and 5% CO_2_. On the next day, horizontal scratches were introduced into the confluent monolayer; the plate was washed thoroughly with PBS, control wells were replenished with fresh medium, while test wells were treated with fresh media containing melanin at 100 μg/ml concentration. Images were taken using an inverted microscope at the indicated time intervals. The plate was incubated at 37°C and 5% CO_2_ in between time points. The taken images were analyzed by MII ImageView software version 3.7.

#### Antimicrobial Activity of the Purified Melanin

The well-cut diffusion technique was applied to test melanin’s antibacterial activity. Various reference strains were used including *S. aureus* ATCC25923, *E. coli* ATCC8739, *Enterococcus faecalis* ATCC29212, *B. subtilis* ATCC6633, and *Aeromonas hydrophila*. With the aid of a sterile cork borer, 5-mm-diameter boreholes were made in nutrient agar plates (10% agar) inoculated individually with bacterial pathogenic strain (optical density = 1), 100 μl of melanin (dissolved in DMSO) were tested, and the desired concentration was added in each well. The clear zone diameter around each well was evaluated in mm upon incubation at 37°C for 24 h (Hassan, 2016).

The antifungal activity of melanin against *Aspergillus niger*, *Aspergillus oryzae*, and *Penicillium* sp. was determined using a modified assay of growth inhibition of the tested fungi (Hassan and Abd El-latif, 2018). At the desired concentration, 100 μl of melanin (dissolved in DMSO) was mixed with sabouraud dextrose agar (SDA). A disk of each indicator fungus (8 mm diameter) was placed on a presterilized SDA medium plate and incubated at 30°C. As a control, a medium devoid of melanin was used. After 72 h, the colony growth diameter was measured and compared with the control. The percentage of fungal growth inhibition was calculated using the equation below:


I=(C-T/C)×100

Where I, percentage of inhibition; C, radial growth in control; T, radial growth in test.

### Environmental Impact

#### Adsorption of Heavy Metals Using Melanin Pigment Entrapped in Alginate Beads

Sodium alginate (3%) was dissolved in water, autoclaved at 121°C for 10 min then left to cool. With the aid of continuous stirring on a magnetic stirrer, 1 mg/ml of pure melanin pigment was added to the sterilized alginate solution. The alginate-melanin mixture was extruded dropwise through a hypodermic needle into a cross-linking solution (sterile 2% CaCl_2_ solution) under steady stirring to obtain spherical beads of calcium alginate gel encasing the pigment. To allow their complete hardening, the beads were left in the calcium solution for about 2 h before being washed several times with sterile distilled water (Sajjan et al., 2013).

Heavy metals such as lead, nickel, and cadmium were removed using previously synthesized melanin beads in an adsorption study. Heavy metal salts were dissolved to produce solutions with final concentrations of 5, 25, 50, and 100 parts per million. One gram of melanin alginate beads was placed in 50-ml conical flasks containing various metal ion solutions, and the flasks were shaken at 100 rpm and kept for 1 h at 37°C. After adsorption of metal ions, the suspensions were centrifuged at 6,000 × *g* for 10 min, and the supernatant was analyzed using flame atomic absorption spectroscopy (contrAA 300 Analytik Jena). FT-IR spectroscopy (PerkinElmer, United States) was used to analyze metal ion-bound melanin-alginate beads that were separated from the metal ion solution (Thaira et al., 2018). The following equation was used to calculate adsorption efficiencies:


Metalremovalefficiency(%)=[(Ic-Fc)×100/Ic]

Where Ic = is the initial concentration (ppm) and Fc = is the final concentration (ppm).

## Results

### Strain Isolation and Identification

In a previous work (Elsayis et al., 2022), five isolates were obtained from local marine habitats. On SDA medium enriched with L-tyrosine and 10% NaCl, all of the acquired isolates were observed to exhibit dark brown to black pigmented colonies and were regarded as probable melanin makers. Also, the generation of melanin by the five isolates was explored. The AS1 isolate (Supplementary Figure 1) among all of them expressed the highest yield of melanin, recording 0.420 g/L; therefore, it was used for further experiments. Furthermore, the AS1 strain was registered in Assiut University Mycological Center under the number AUMC 14501, and molecularly recognized as *Hortaea werneckii* AS1. The nucleotide sequence was assigned under the accession number MW187022 in the GenBank sequence database.

### Melanin Extraction and Characterization

From the harvested yeast biomass, melanin pigment was recovered as described by Gadd (1982; Supplementary Figures 2A–C), and the identification of the pigment using physiochemical characterization, UV-Vis spectrophotometry, FTIR, elemental analysis, and SEM claimed that it was a eumelanin pigment (Elsayis et al., 2022).

### Biological Impact

#### Evaluation of Antioxidant Activity of *Hortaea werneckii* AS1

Using a procedure based on the decolorization of the stable-colored radical DPPH, the antioxidant potential of the extracted melanin pigment has been investigated (Ben Tahar et al., 2019). The initial screening step revealed that the IC_50_ value of the tested melanin was found to be below 100 μg/ml. Therefore, the following concentrations were prepared and tested in the same way: 80, 60, 40, 20, and 10 μg/ml. The results revealed that melanin’s IC_50_ value was recorded at 61.38 ± 4.72 μg/ml, and was 10 times higher than trolox as a standard (6.112 μg/ml).

#### Assessment of the Purified Melanin Cytotoxicity

**FIGURE 1:**
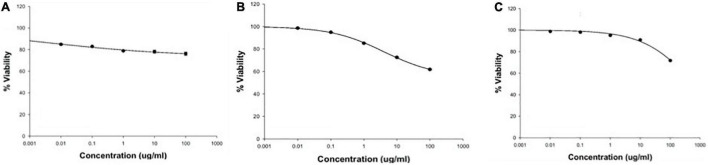
Cell viability percentage of melanin’s cytotoxicity at concentrations up to 100 μg/ml on **(A)** human skin fibroblast cell line (HSF), **(B)** hepatocellular carcinoma cell line (HepG2), and **(C)** breast adenocarcinoma cell line (MCF-7).

Sulforhodamine B assay was carried out on a normal (HSF) cell line using various concentrations (0.01, 0.1, 1, 10, and 100 μg/ml) of melanin, and cell viability was calculated. As shown in Figure 1A, the highest reduction in cell viability (23.8%) was recorded at 100 μg/ml melanin concentration, implying that there is no considerable lethal impact on cell’s metabolism. The IC_50_ of melanin on the tested cell line (HSF) was greater than 0.1 mg/ml, which is supposed to be the settled border of toxicity to human cell lines for natural compounds (Oonsivilai et al., 2008). Therefore, *H. werneckii* AS1 melanin is biocompatible and can be adopted as a natural antioxidant with various cosmetics and pharmaceutical applications.

In addition, the purified melanin pigment’s anticancer activity was investigated on two cancer cell lines: HepG2 and MCF-7. Cell viability was 61.2 and 71.68% for HepG2 and MCF-7, respectively, at a concentration of 100 μg/ml melanin (Figures 1B,C). However, the tested pigment exerts a reduction percentage of approximately 39% on the HepG2 cell line; these results indicated that *H. werneckii* AS1 melanin is a poor anticancer agent with an IC_50_ value of more than 100 μg/ml.

#### *In vitro* Wound Healing Assay

**FIGURE 2:**
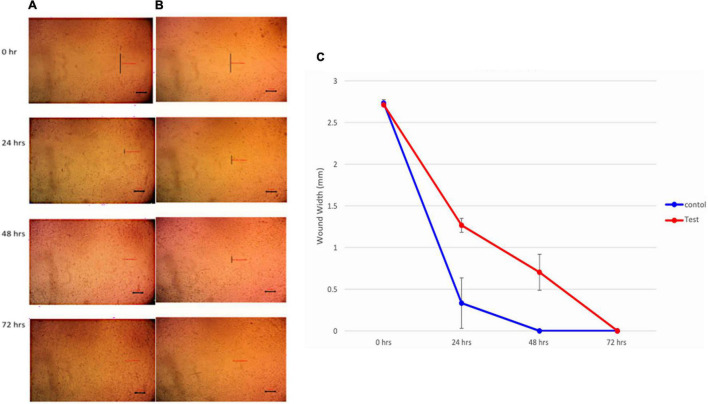
Images of scratch closure rate **(A)** control without melanin, **(B)** test with 100 μg/ml melanin, and **(C)** wound width calculated as the distance between the edges of the scratches at various time intervals.

*Hortaea werneckii* AS1 wound healing activity was assessed by scratch assay using 100 μg/ml melanin. In dermatology, it is a regularly used approach for determining the toxicity of a biological compound (Grada et al., 2017). The scratch of HSF cells was entirely closed either in the presence (test) or in the absence (control) of melanin after 72 h of incubation (Figures 2A–C). These findings showed that melanin at a concentration of 100 μg/ml has no influence on cell migration.

#### Antimicrobial Activity of the Purified Melanin

**FIGURE 3:**
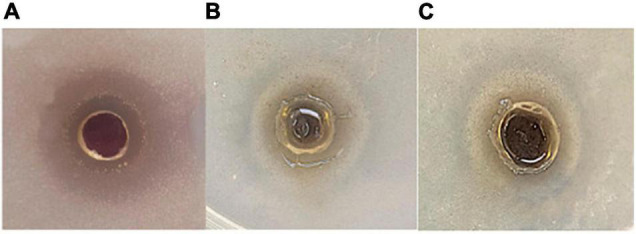
Antimicrobial effect of *H. werneckii* AS1 melanin using agar diffusion method on **(A)** *Aeromonas* sp., **(B)** *Staphylococcus aureus*, and **(C)** *Enterococcus faecalis*.

Using agar well diffusion method (Valgas et al., 2007), melanin’s antibacterial activity was evaluated at melanin concentrations of 50, 100, and 150 μg/ml. The pigment showed antimicrobial activity against Gram-positive bacteria *E. faecalis* and *S. aureus* with a zone of inhibition of 15 and 20 mm at melanin concentrations of 50 and 150 μg/ml, respectively, but showed no effect against tested *B. subtilis* strain. It also exhibited an inhibiting effect on the tested Gram-negative *A. hydrophila* at a melanin concentration of 150 μg/ml and inhibition zone of 25 mm, but without antibacterial activity against *E. coli* (Figure 3).

These findings indicated that melanin is more effective against Gram-negative bacteria than Gram-positive strains. Gram-negative bacteria are known to be more antibiotic resistant owing to their distinct outer membrane and associated lipopolysaccharide molecules that discriminate them from Gram-positive strains (Breijyeh et al., 2020). Furthermore, the sensitivity of *A. hydrophila* toward *H. werneckii* AS1 melanin suggests the pigment’s incorporation in biological control in aquaculture since *A. hydrophila* is a famous pathogen linked to high mortality in aquaculture (Zhou et al., 2019).

**FIGURE 4:**
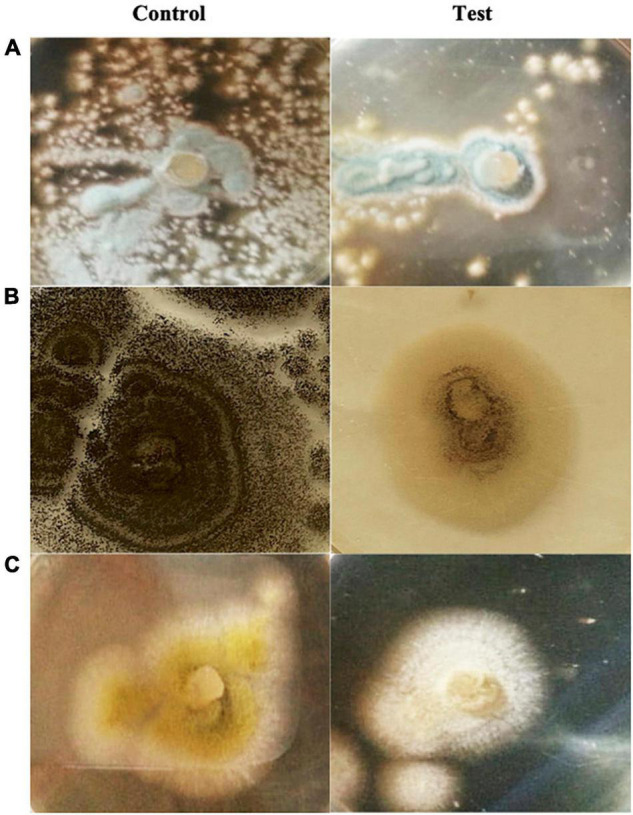
Growth inhibition and antifungal effect of *H. werneckii* AS1 melanin at a concentration of 100 μg/ml on **(A)** *Penicillium* sp., **(B)** *Aspergillus niger*, and **(C)** *Aspergillus oryzae*.

The antifungal activity of melanin was tested against *A. niger*, *A. oryzae*, and *Penicillium* sp. at 100 μg/ml, and the inhibition percentage results were 63.6, 52.9, and 58.8%, respectively. Also, these results suggested that melanin has a significant antifungal potential against the tested strains (Figure 4).

### Environmental Impact

#### Adsorption of Heavy Metals Using Melanin Pigment Entrapped in Alginate Beads

Adsorption capacity of sodium alginate entrapped melanin pigment was studied using lead, nickel, and cadmium heavy metals. Under the tested conditions (see section “Materials and Methods”), and as shown in Table 1, at 100 mg/L metal concentration, melanin showed a considerable adsorption affinity toward Pb^2+^, Cd^2+^, and Ni^2+^ with a removal efficiency of 85.7, 84.8, and 81.5%, respectively. Also, it can be noticed that the removal efficiency is exponentially related to the metal concentration. In spite of the lower efficiency at lower concentrations (5 mg/L), melanin-alginate beads showed a good level of metal withdrawal up to 70% regarding to Cd^2+^.

**TABLE 1:** Removal efficiency and adsorption capacity of melanin for Ni^2+^, Pb^2+^, and Cd^2+^.

Conc. (mg/l)	Ni^2+^	Pb^2+^	Cd^2+^
	Removal efficiency (%)	Removal efficiency (%)	Removal efficiency (%)
5	61.4%	50.0%	70.6%
25	62.4%	51.6%	71.0%
50	66.6%	77.6%	72.5%
100	81.5%	85.7%	84.8%

**FIGURE 5:**
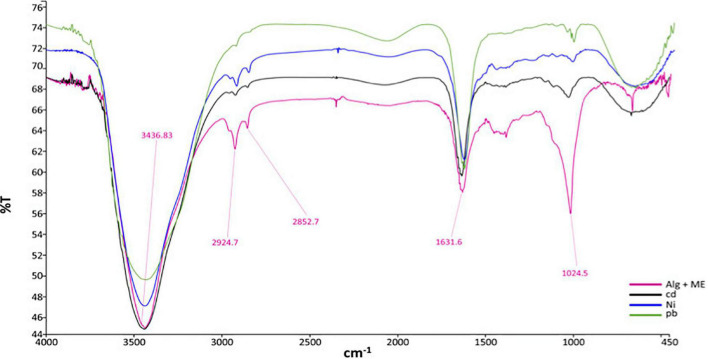
FTIR spectra of (pink) alginate entrapped melanin, (blue) nickel-bound melanin, (black) cadmium-bound melanin, and (green) lead-bound melanin.

The IR spectrum of melanin entrapped in alginate beads, according to Figure 5, indicates a number of the significant peaks linked with the pigment. -OH and -NH stretching caused the broad band at 3,436.83 cm^–1^. Also, the tiny assorted bands nearby 2,924.7 cm^–1^ and 2,852.7 cm^–1^ were ascribed to -CH groups stretching and for N-H stretches, respectively. The waves of the aromatic groups (-C=O or -C=C) have a notable absorption peak at 1,631.6 cm^–1^. Finally, the aromatic ring -CH broadening is responsible for the absorption peak noticed at 1,024.6 cm^–1^ (Kumar et al., 2011; Yao et al., 2016).

The peak intensities at 2,924.7 cm^–1^ and 2,852.7 cm^–1^, as well as 1,024.6 cm^–1^, were reduced following exposure to Pb^2+^, Cd^2+^, and Ni^2+^ metals, according to results shown in Figure 5, indicating that -NH groups are possible binding sites for the tested metals. In addition, the increase in wave intensities at 668 cm^–1^ and 670 cm^–1^ after adsorption of metals is a further sign of strong interaction between the -CH group and the metal ions (Thaira et al., 2018).

## Discussion

Melanin is an attractive pigment with physiochemical characteristics that make it suitable for incorporation in a range of medical practices. Melanin has the property of receiving or donating an electron due to the unpaired electrons contained in its structure, and as a result, it may easily interact with free radicals, acting as an antioxidant (El-Naggar and El-Ewasy, 2017; Wang and Rhim, 2019). The IC_50_ value is the criterion used to assess the antioxidant activity of the test pigment sample. Generally, IC_50_ is defined as the concentration required to reduce the original DPPH concentration by 50%; consequently, the lower the IC_50_ value, the strong the antioxidant activity (Rivero-Cruz et al., 2020). In the DPPH radical scavenging experiment, *H. werneckii* AS1 melanin recorded an IC_50_ value of 61.38 μg/ml. Such a result (with an IC_50_ value below 100 μg/ml) was in the range of values recorded by the purified melanin pigment from *Aspergillus bridgeri*, with a radical scavenging activity IC_50_ of 54.12 μg/ml (Kumar et al., 2011), as well as a eumelanin pigment from *Pseudomonas putida* ESACB191 with IC_50_ at 74 μg/ml, compared to 170.3 μg/ml for commercial *Sepia officinalis* melanin (Ferraz et al., 2021). Furthermore, the IC_50_ of *H. werneckii* R23 melanin on DPPH radicals was 28.11 ± 10.76 μg/ml, which was 21 times beyond the standard trolox IC_50_ of 1.334 ± 0.20 μg/ml (Wilson, 2016). On the other hand, our results showed a higher antioxidant activity than melanin extracted from the yeast *Yarrowia lipolytica* W29, which showed an IC_50_ value of 230 μg/ml, against DPPH (Ben Tahar et al., 2019). Also, melanin extracted from the fruiting bodies of *Auricularia auricula* has an antioxidant effect with an IC_50_ of 180 μg/ml (Zou et al., 2015).

The previously mentioned data suggested that melanin had a significant property of scavenging free radicals at various scales. The fact that melanin has a complex structure of various compounds with hydroxyl and aromatic groups that can easily be stabilized through donating hydrogen to the DPPH radical explains its high antioxidant activity. Natural antioxidants are recommended for use in food, cosmetic, and pharmaceutical industries (Kumar et al., 2011; Ferraz et al., 2021). Therefore, *H. werneckii* AS1 can be recommended as a promising natural antioxidant due to its considerable antioxidant potential.

The assessment of *H. werneckii* AS1 melanin’s cytotoxicity toward mammalian cells is a must before using it as an ingredient in medical and pharmaceutical applications. *H. werneckii* AS1 melanin was shown to be non-cytotoxic, with an IC_50_ greater than 100 μg/ml. Similarly, but at a higher concentration, purified melanin from *Dietzia schimae* NM3 at a concentration of up to 250 μg/ml showed no toxicity toward normal human fibroblast cell culture (HFB) (Eskandari and Etemadifar, 2021). Also, *P. putida* ESACB 191 melanin tested against A375, HeLa Kyoto, HepG2, and Caco2 cell lines, showed an elevated IC_50_ values of 1.77, 2.51, 0.89, and 1.08 mg/ml, respectively (Ferraz et al., 2021). Furthermore, melanin from *Y. lipolytica* W29 was found to exert no toxic effect on mouse fibroblast NIH3T3 and human keratinocyte HaCaT cell lines with a 20% reduction of the metabolic activity of the cells at a concentration of 100 μg/ml (Ben Tahar et al., 2019). In contrast to previously mentioned results, against normal human lung fibroblast (WI-38) and human amnion cells (WISH), *Streptomyces glaucescens* strain NEAE-H-purified melanin pigment had an IC_50_ value of 37.05 and 48.07 μg/ml, respectively (El-Naggar and El-Ewasy, 2017).

In terms of the effect of *H. werneckii* AS1 melanin pigment on cancerous cell lines, a mild effect was detected at a concentration of 100 μg/ml on the malignant HepG2 cell line, with a reduction percentage of roughly 39%. These findings seem to be in contrast with the results gained by Shalaby et al. (2019) who stated that using SRB cytotoxicity assay, *Bacillus licheniformis* MAL black eumelanin exhibited an encouraging anticancer action against human hepatocellular carcinoma (HepG2) and colon carcinoma (HCT-116) cell lines with an IC_50_ rate of 6.15 and 5.54 μg, respectively, in comparison with Doxorubicin, which had IC_50_ values of 4.05 and 4.45 μg (Shalaby et al., 2019). Furthermore, Kurian et al. (2015) documented that marine *Bacillus* spp. BTCZ31 melanin reduced the proliferation of the L929 cell line using the MTT assay, with an IC_50_ of 105.4 μg/ml.

Moreover, *H. werneckii* AS1 melanin was recognized to exhibit no detrimental effect toward tissue regeneration of human skin fibroblast (HSF) cells at a dosage of 100 μg/ml. In relevance with these results, the wound healing assay of *Y. lipolytica* W29 pyomelanin examined on NIH3T3 and HaCaT cell lines showed a scratch closure close to 100% after 48 h. There was no documented difference in the rate of cell migration in cells exposed to pyomelanin at 100 μg/ml in comparison to non-exposed cells (Ben Tahar et al., 2019). On the other hand, other reports have documented the melanin considerable healing impact on wounds due to its antioxidant and antibacterial action in cosmetic formulations (Taburets et al., 2016; Poulose et al., 2020).

The antimicrobial capability of *H. werneckii* AS1 melanin pigment was relevant against various microorganisms. Some of our results go along with Barretto and Vootla’s (2020) work on melanin from *Cryptococcus rajasthanensis* KY627764 associated with the gut of *Bombyx mori* at concentrations of 25–100 μg/ml, which was documented to have antimicrobial activity against *S. aureus, B. subtilis, E. coli*, and *P. aeruginosa* with inhibition zone diameters of 10, 16, 12, and 18 mm, respectively. It also showed antifungal activity when tested against *C. albicans* at concentrations ranging from 25 to 100 μg/ml, with a 4- to 20-mm inhibition zone recorded. Furthermore, Rani et al. (2013) reported that the antimicrobial activity of melanin from the halophilic black yeast *H. werneckii*, which had inhibitory potential against *Salmonella typhi* (17 mm) and *Vibrio parahaemolyticus* (15 mm). Following the same path, melanin from *Halomonas venusta* infused with seaweed concentrate inhibited *S. aureus* and *S. pyogenes* (Poulose et al., 2020). Moreover, as reported in our results, Fitrial and Khotimah (2021) documented the bactericidal effect of cuttlefish ink melanin on *Aeromonas* sp. that was manifested by the shrinkage in cell size as well as the deformation in cell shape. In contrast to our findings, melanin from *Aspergillus bridgeri* ICTF-201, had no antimicrobial activity against any Gram-positive or Gram-negative bacteria, including *B. subtilis*, *Micrococcus luteus*, *S. aureus*, *E. coli*, *Klebsiella planticola*, and *P. aeruginosa*, as well as *C. albicans* (Kumar et al., 2011).

The outcome of this batch of tests pointed out that *H. werneckii* AS1 melanin is non-toxic to the mammalian cell line examined, especially under the conditions applied. Their antioxidant, antibacterial, and antifungal properties, as well as the fact that they have no impact on skin wound’s regeneration, support its use in a variety of cosmetic and medicinal formulations.

In addition, *H. werneckii* AS1 melanin manifested a reasonable chelating capacity toward Pb^2+^, Cd^2+^, and Ni^2+^ metal ions (at a concentration of 5 and 100 mg/L) when used as a biosorbent. Similar to these results, melanin extracted from squid *Ommastrephes bartrami* were used for biosorption of Cd^2+^ and Pb^2+^ at a concentration of 2 mM/L recording 95% adsorption efficiency, and the highest yield and stability were obtained within a pH range of 4.0–7.0 (Chen et al., 2009). Thaira et al. (2018) observed that above pH 4, melanin has negatively charged functional groups such as the carboxyl group, which attract positively charged cations, and thus, melanin from *Pseudomonas stutzeri* strain HMGM-7 under optimum conditions showed adsorption efficiency of lead, chromium, and selenium of 97, 88.9, and 82%, respectively. In addition, melanin from *Bacillus safensis* showed an affinity to capture ferrous ions with a maximum effect of about 64% chelation in a solution of 2 mM FeCl_2_ (0.05 ml) (Tarangini and Mishra, 2014).

After the adsorption process, FTIR spectra of melanin entrapped in alginate beads exhibited a vibration in peak intensities at 2,924.7 cm^–1^, 2,852.7 cm^–1^, 1,024.6 cm^–1^, as well as 668 cm^–1^, and 670 cm^–1^. In harmony with previously mentioned results, during selenium adsorption using free melanin from *P. stutzeri* strain HMGM-7, peaks with wavelengths corresponding to 639.21 cm^–1^ and 676.15 cm^–1^ increased in intensity as an indication for -CH bond vibrations. Moreover, two other peaks with increased intensity at 837.64 cm^–1^ and 1,062.31 cm^–1^ were detected as an indication of selenium binding to NH group of melanin (Thaira et al., 2018). Some different results away from those previously mentioned were obtained during the immobilization of melanin pigment from *Klebsiella* sp. GSK for the adsorption of copper and lead ions. In case of Cu^2+^ ions, with the aid of FTIR spectroscopy, it was noticed that the functional groups for its binding were -OH and -NH groups, which was manifested by the shifting in their markable peak intensity from 3,445.18 cm^–1^ to 3,432.20 cm^–1^. Moreover, the bond between Cu^2+^ and hydroxyl groups was indicated through reduction in the -C = O peak from 1,637.57 cm^–1^ to 1,633.47 cm^–1^. The same notes were illustrated regarding Pb^2+^ ions, in addition to the peak shift from 1,424.60 cm^–1^ to 1,412.84 cm^–1^ showing the binding of ions to Pb^2+^ aromatic -C = C (Shrishailnath et al., 2010).

## Conclusion

Melanin pigment extracted from marine halotolerant black yeast *H. werneckii* AS1 was assessed for its antioxidant activity using 2,2-diphenyl-1-picrylhydrazyl (DPPH) radical scavenging assay and recorded a promising IC_50_ value of 61.38 ± 4.72 μg/ml, suggesting its usage as a natural antioxidant. This funding was supported with the fact that melanin pigment undergoes the cytotoxicity test successfully with only 23.8% reduction in the cell viability of human skin fibroblast cell line (HSF) at a melanin concentration of up to 100 μg/ml. In addition, the pigment showed an antibacterial activity toward *S. aureus* and *A. hydrophila* with inhibition zone diameters of 20 and 25 mm, respectively, at 150 μg/ml pigment concentration. Hence, it can further be applied as an antimicrobial agent against fatal pathogenic strains affecting the aquaculture industry. Regarding melanin environmental application, heavy metal removal was tested using entrapped melanin in alginate beads. Melanin showed high affinity toward Cd^2+^ with a removal efficiency of approximately 84.8% at 100 ppm of metal concentration, encouraging its use as an eco-friendly absorbent for wastewater treatment. Hence, *H. werneckii* AS1 melanin is an attractive harmless candidate for various medical and environmental applications.

## Data Availability Statement

The datasets presented in this study can be found in online repositories. The names of the repository/repositories and accession number(s) can be found below: https://www.ncbi.nlm.nih.gov/, MW187022.

## Author Contributions

AE, HK, SH, and KG conceived and designed the research. AE conducted the experiments. AE, SH, and HK contributed to analyzing the data. AE wrote the manuscript. All authors revised the manuscript, and finalized and agreed to the final version of the manuscript.

## Conflict of Interest

The authors declare that the research was conducted in the absence of any commercial or financial relationships that could be construed as a potential conflict of interest.

## Publisher’s Note

All claims expressed in this article are solely those of the authors and do not necessarily represent those of their affiliated organizations, or those of the publisher, the editors and the reviewers. Any product that may be evaluated in this article, or claim that may be made by its manufacturer, is not guaranteed or endorsed by the publisher.

## Abbreviations

DPPH: 2,2-diphenyl-1-picrylhydrazyl
HSF: human skin fibroblast cell line
ROS: reactive oxygen species
rpm: round per minute
UV-Vis: ultraviolet-visible spectrophotometry
FTIR: Fourier transform-infrared spectroscopy
SEM: scanning electron microscopy
DMSO: dimethyl sulfoxide
SRB: sulforhodamine B assay
HepG2: hepatocellular carcinoma cell line
MCF-7: breast adenocarcinoma cell line
DMEM: Dulbecco’s Modified Eagle medium
TCA: trichloroacetic acid
PBS: phosphate buffered saline
SDA: sabouraud dextrose agar.
