# Supplementary material for: Suggested Sustainable Medical and Environmental Uses of Melanin Pigment From Halotolerant Black Yeast Hortaea werneckii AS1
Source: Front Microbiol. 2022 Apr 13;13:871394. doi: 10.3389/fmicb.2022.871394 (PMC9048979; doi:10.3389/fmicb.2022.871394)
Supplement: Supplementary file 1 [file Data_Sheet_1.pdf]

Supplementary Material

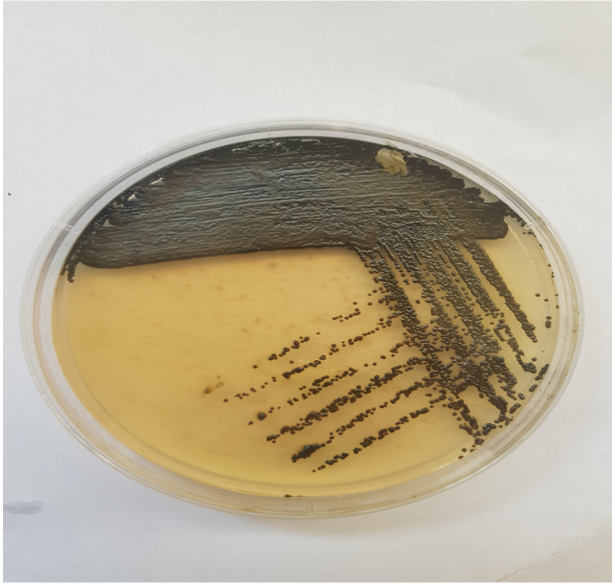

Figure 1. *Hortaea werneckii* AS1 melanized colonies on Sabouraud Dextrose Agar (SDA)

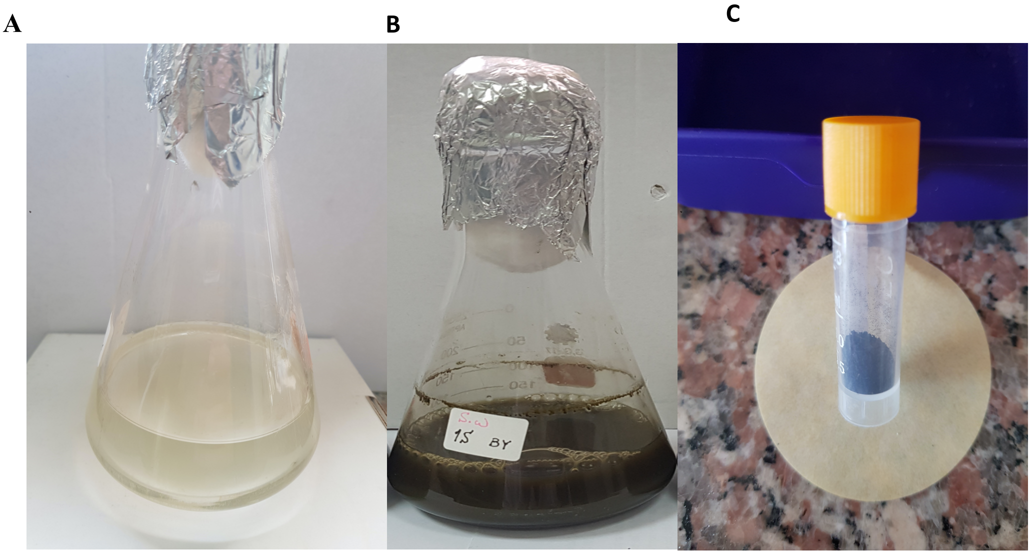

Figure 2. **(A)** Control culture of modified Vogel liquid medium, **(B)** a grown culture of *Hortaea werneckii* AS1 in modified Vogel liquid medium prepared with seawater, and **(C)** extracted melanin pigment from yeast biomass.
